# Assessing alcohol consumption across phosphatidylethanol levels using HDL-cholesterol as a predictor

**DOI:** 10.1093/alcalc/agae085

**Published:** 2024-12-13

**Authors:** Alexander Årving, Thor Hilberg, Elisabeth Wiik Vigerust, Benedicte Jørgenrud, Stig Tore Bogstrand, Jørg Mørland, Gudrun Høiseth

**Affiliations:** Department of Forensic Sciences, Oslo University Hospital, P.O. Box 4950 Nydalen, Oslo 0424, Norway; Fürst Medisinsk Laboratorium, P.O. Box 158 Alnabru, Oslo 0614, Norway; Fürst Medisinsk Laboratorium, P.O. Box 158 Alnabru, Oslo 0614, Norway; Department of Forensic Sciences, Oslo University Hospital, P.O. Box 4950 Nydalen, Oslo 0424, Norway; Department of Forensic Sciences, Oslo University Hospital, P.O. Box 4950 Nydalen, Oslo 0424, Norway; Department of Public Health Science, Institute of Health and Society, Faculty of Medicine, University of Oslo, P.O. Box 1089 Blindern, Oslo 0318, Norway; Norwegian Institute of Public Health, P.O. Box 222 Skøyen, Oslo 0213, Norway; Norwegian Centre for Addiction Research (SERAF), Institute of Clinical Medicine, Faculty of Medicine, University of Oslo, P.O. Box 1039 Blindern, Oslo 0315, Norway; Department of Forensic Sciences, Oslo University Hospital, P.O. Box 4950 Nydalen, Oslo 0424, Norway; Norwegian Centre for Addiction Research (SERAF), Institute of Clinical Medicine, Faculty of Medicine, University of Oslo, P.O. Box 1039 Blindern, Oslo 0315, Norway; Center for Psychopharmacology, Diakonhjemmet Hospital, P.O. Box 23 Vinderen, Oslo 0319, Norway

**Keywords:** alcohol biomarker, phosphatidylethanol, HDL-C, alcohol consumption

## Abstract

**Aims:**

Prior research has established a correlation between increases of High-Density Lipoprotein Cholesterol (HDL-C) levels and alcohol consumption. This study aimed to explore the association between phosphatidylethanol (PEth) levels and the amount of consumed ethanol, utilizing HDL-C as a surrogate marker on a population level. This endeavor offers an adjunct to other studies.

**Methods:**

PEth and HDL-C levels in 50 751 samples from 29 899 patients in Norway were measured simultaneously in whole blood and serum, respectively. Linear mixed model analyses were employed to assess HDL-C levels within different PEth intervals. Drawing on previous research indicating an increase of .0035 mmol/L in HDL-C per gram of pure ethanol consumed per day, and assuming no alcohol intake in the zero PEth group, we estimated mean daily ethanol intake at the group level for males in each PEth interval.

**Results:**

Results revealed a significant correlation between PEth and HDL-C levels (Spearman’s rho = .385 for women, .420 for men, *P <* .001). Estimated mean HDL-C levels indicated higher alcohol consumption with increasing PEth. Specifically, men with PEth values in the .031–0.100 μmol/L (22–70 ng/ml) interval were estimated to consume approximately mean 20 grams of ethanol daily, while those in the .301–0.500 μmol/L (212–351 ng/ml) PEth interval had an estimated mean daily ethanol intake of 51 grams.

**Conclusions:**

The results from this study suggest an approximate estimation of mean daily amounts of consumed ethanol at group levels in different PEth intervals, based on previously shown correlation of ethanol consumption and HDL-C increase.

## Teaser

**Short Summary:** This study found a significant correlation between phosphatidylethanol (PEth) and high density lipoprotein-cholesterol (HDL-C) levels. Mean estimated daily ethanol consumption increased with higher PEth intervals, with 20 grams for PEth .031–0.100 μmol/L and 51 grams for PEth .301–0.500 μmol/L, using HDL-C at the group level for males as a predictor.

## Introduction

Phosphatidylethanol (PEth) has emerged as a prevalent biomarker for alcohol consumption, steadily gaining popularity and overshadowing the traditional marker, Carbohydrate-Deficient Transferrin (CDT), commonly used for this purpose (Helander and Hansson 2023). Unlike CDT and other indirect alcohol markers, PEth provides a direct reflection of cumulative alcohol intake in a given individual and is less influenced by confounding factors such as medical conditions or genetic variations (Schröck et al. 2014). In a former study, we concluded that PEth seemed to be a more sensitive and reliable biomarker for assessing alcohol consumption compared to CDT (Årving et al. 2021). PEth concentrations exceeding .3 μmol/L (~211 ng/ml) are generally indicative of harmful levels of alcohol consumption, while levels below .03 μmol/L (~21 ng/ml) typically signify abstinence or low alcohol intake (Luginbühl et al. 2022). Values falling within these thresholds are often interpreted as indicative of moderate alcohol consumption (Aakerøy et al. 2016; Ulwelling and Smith 2018; Helander and Hansson 2023).

Long-standing epidemiological research has indicated a potential protective effect of light to moderate alcohol consumption against medical conditions such as myocardial infarction and type 2 diabetes. However, emerging research suggests that establishing a safe lower limit for alcohol intake, particularly concerning the risk of various types of cancer, may be challenging. Total abstinence seems to be associated with the lowest mortality risk among young adults. At the same time, moderate alcohol consumption in older age groups does not appear to significantly increase overall mortality (GBD 2020 Alcohol Collaborators 2022; Thelle and Grønbæk 2024).

Given the evolving understanding of alcohol as a health risk factor, it could prove beneficial for clinicians to establish a connection between measured PEth levels and the corresponding amount of alcohol consumed, expressed in grams or alcohol units, over a specific timeframe. Such an approach would contribute to a more nuanced understanding of the implications of PEth measurements in alignment with contemporary guidelines and research findings.

The optimal study design for examining ethanol consumption across various PEth intervals would ideally involve experimental studies conducted under controlled conditions. However, the ethical implications arising from the necessity of subjects consuming relatively high amounts of ethanol over an extended time period may pose challenges. Self-reported consumption stands as an alternative approach, though it is susceptible to under-reporting biases (Dawson 2003; Magnus et al. 2011; Klatsky et al. 2014; Livingston and Callinan 2015). Consequently, alternative methodologies should also be explored.

Meta-analyses of experimental studies have found a dose–response relationship between increased alcohol consumption and changes of high density lipoprotein-cholesterol (HDL-C) (Rimm et al. 1999; Brien et al. 2011). Specifically, an elevation of .0035 mmol/L (.134 mg/dl) in serum HDL-C among men was reported by Rimm et al. (1999) for each gram increase of pure ethanol consumed per day. This meta-analysis encompassed data from 29 records that exclusively studied men, and only three records that exclusively studied women. Consequently, the relationship between changes in HDL-C levels and alcohol consumption is more extensively documented for male individuals.

To complement other investigative methods, the levels of HDL-C could be employed as a surrogate measure to estimate actual mean ethanol consumption on group levels (Magnus et al. 2011). This strategy may offer additional insights into the relationship between ethanol intake and HDL-C levels, serving as a valuable adjunct to other research approaches.

In the present study, our objective was to investigate the quantity of ethanol ingested by patients with varying PEth levels, utilizing HDL-C increase as a predictive marker at group level for actual consumption.

## Material and methods

### Data collection

The data for this study were derived from analyses of PEth 16:0/18:1 and HDL-C conducted in Norway at the Fürst Medisinsk Laboratorium between September 2016 and November 2023. The study database comprised anonymous and encrypted information, including age, sex, sampling dates, and analytical results. Samples were predominantly collected from patients attending primary care physicians, with some originating from social care institutions. Unfortunately, additional details about the characteristics of the study population were unavailable. Inclusion criteria necessitated simultaneous measurements of both PEth (in whole blood) and HDL-C (in serum) for individual patients. Some of the serum samples additionally underwent analyses for ethanol and/or other biomarkers including Carbohydrate-Deficient Transferrin (CDT), Aspartate Aminotransferase (AST), Alanine Aminotransferase (ALT), and Gamma-Glutamyl Transferase (GGT).

### Sample preparation

Serum and EDTA-anticoagulated whole blood samples were obtained by drawing venous blood into the Serum SST or EDTA-prefilled vacutainer tubes, respectively (both from Beckton Dickinson Norway, Oslo Norway) with further processing according to manufacturer’s instructions. All analytes were measured within the same day upon arrival to the laboratory.

### Analyses for PEth and CDT

Analyses for PEth and CDT were conducted as described in detail in a previous publication (Årving et al. 2021). In brief, whole blood was analyzed for PEth using a Waters Acquity UPC2 (TM) Ultra Performance Convergence chromatography system connected to Waters TQ-S triple quadrupole mass-spectrometer (UPC2-MS/MS) (Waters, Milford, MA, USA). The limit of quantification (LoQ) was .015 μmol/L (11 ng/ml). Concentrations below this level were set to zero. Serum CDT was quantified by electrophoretic separation of the transferrin fractions using a classic Sebia Capillarys 2 (Lisses, France) without CDTIFCC standardization. The LoQ was .4 percentage points for CDT.

### Analyses for HDL-C, AST, ALT, GGT, and EtOH

Analyses for HDL-C, AST, ALT, GGT, and EtOH were conducted on non-fasting serum samples utilizing the Advia Chemistry XPT (Siemens Healthineers, Erlangen, Germany). Reference levels were established in accordance with The Nordic Reference Interval Project (Rustad et al. 2004).

HDL-C was measured in serum without prior separation (Izawa et al. 1997). Cholesterol from non-HDL particles was released and eliminated during the first step of the reaction. Cholesterol in HDL particles was released during the second step of detergent in the D-HDL reagent 2, and HDL-C was measured using a Trinder reaction. The assay was linear from .2 to 3 mmol/L for serum. Results below the lower limit of the analytical range were reported as <.2 mmol/L. An automated rerun function extended the upper limit to 6 mmol/L. The analytical coefficient of variation was 2.1%.

### Calculations

The material was categorized based on established PEth interpretation thresholds of ≤.03 μmol/L (~21 ng/ml) representing abstinence or low alcohol intake, and > .3 μmol/L (~211 ng/ml) indicative of heavy consumption (Helander and Hansson 2013; Aakerøy et al. 2016; Ulwelling and Smith 2018). Additionally, PEth concentrations were further subdivided into narrower intervals for possible quantification of alcohol consumption levels. In the group of patients with PEth values of zero (<.015 μmol/L, <11 ng/ml), it was assumed that there was zero consumption of ethanol. A conversion factor for PEth of 1 μmol/L = 703 ng/ml was used, based on molecular weight of PEth 16:0/18:1.

From the 29 data records in the studies examining the correlation between changes of HDL-C levels and ethanol intake in males, a mean increase of .0035 mmol/L in HDL-C per gram of ethanol consumed per day was observed (Rimm et al. 1999). Consequently, the difference in male HDL-C levels between each PEth interval and the zero PEth group was divided by .0035 mmol/L to estimate the mean ethanol consumption for all PEth intervals. Given the limited documentation regarding the relationship between HDL-C levels and ethanol consumption in females, these computations were exclusively conducted for males in the current investigation.

### Statistics

SPSS IBM SPSS Software version 29.0 was utilized for statistical analysis of the data. Normality of PEth and HDL-C levels was assessed with histograms and Q-Q plots and tested using the Kolmogorov–Smirnov method, indicating a deviation from normal distribution (*P <* .001). The correlation between PEth and HDL-C levels was examined using Spearman’s correlation coefficient. Linear mixed model analyses (using random intercept and the restricted maximum likelihood model) were used to allow for inclusion of multiple samples per patient when assessing mean HDL-C concentration across various PEth intervals.

### Ethics

Ethical approval was obtained from Regional Committee for Medical and Health Research Ethics, Region South-East, Norway (2018/1041). Due to the large size of the data material and the anonymous handling of the data, the study was approved to be performed without informed consent from each of the participants.

## Results

In total, 50 751 samples from 29 899 unique patients were included in the study after excluding cases with HDL-C concentrations below .2 mmol/L (N = 258) or at 6 mmol/L or higher (N = 1). The distribution of the number of samples, number of patients, age, and sex for the total material and the different PEth groups are presented in Table 1.

**Table 1:** Distribution of samples and number of patients, along with age and sex information in the total material and in distinct PEth intervals. Conversion factor for PEth: 1 μmol/L = 703 ng/ml

PEth intervals (μmol/L)	PEth intervals converted to ng/ml	No. of samples	No. of patients	% male samples	Age, mean (SD), years
Total		50 751	29 899	68	56.1 (15.3)
<0.015	<11	12 297	9215	64	52.9 (16.8)
0.015–0.030	11–21	2865	2627	66	52.6 (17.1)
0.031–0.100	22–70	6294	5320	68	54.2 (16.7)
0.101–0.200	71–140	5710	4660	70	56.4 (15.4)
0.201–0.300	141–211	4200	3487	71	57.6 (14.7)
0.301–0.500	212–351	5466	4307	70	59.0 (13.9)
0.501–1	352–703	6762	4990	70	59.1 (13.1)
>1	>703	7157	4730	68	58.6 (11.7)

**Figure 1:**
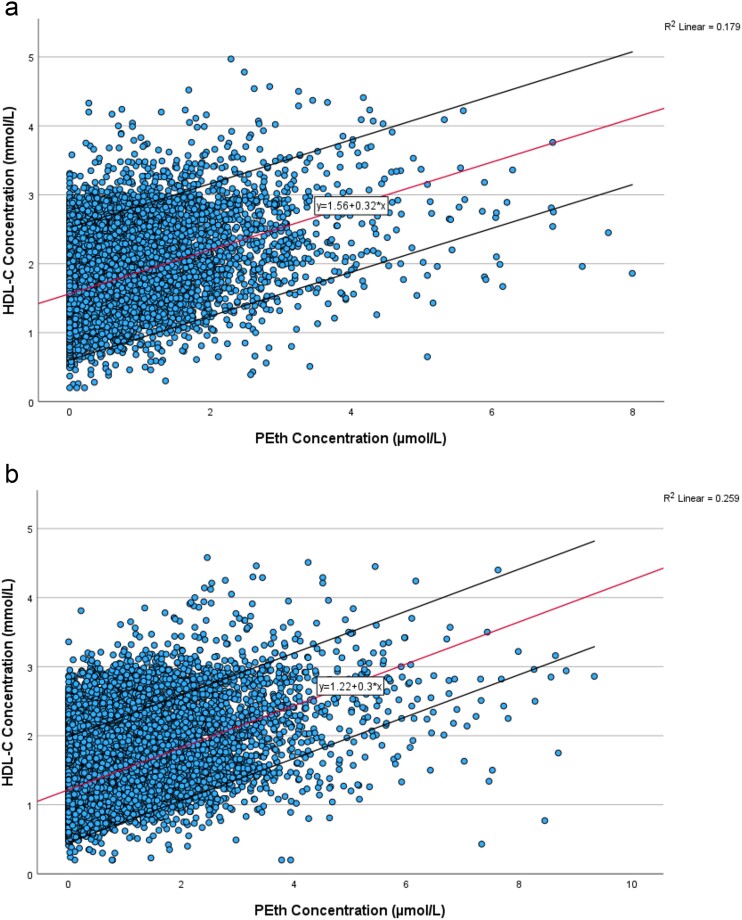
Scatterplots showing the relationship between HDL-C concentration and PEth concentration among females (a) and males (b), respectively. The data points represent individual measurements, while the middle line represents the linear trendline fitted to the data. The upper and lower lines represent the 95% confidence interval for the trendline, based on individual data points. The equation of the trendline is provided as: Y = a + bX, where Y represents HDL-C concentration, X represents PEth concentration, and a and b are coefficients derived from the linear regression analysis.

Figure 1 illustrates the general association between PEth concentration and HDL-C concentration across all samples, with separate plots for females (1a) and males (1b).

The linear regression equations, *Y = 1.56 + .32X* for females and *Y = 1.22 + .30X* for males, thus suggest an approximate increase of .3 mmol/L in HDL-C for every 1 μmol/L increase in PEth concentration. Correlation analysis revealed Spearman’s rho values for the relation between PEth and HDL-C of .385 for females and .420 for males, both demonstrating statistical significance (*P <* .001).

In the total material, 62% of the samples were additionally analyzed for AST, 89% for ALT, 74% for GGT and 35% for CDT. Table 2 shows the percentage of male cases with elevated levels of these biomarkers as well as the mean CDT levels in different PEth groups.

**Table 2:** Percentage of male samples with measured values above age adjusted reference ranges for AST, ALT, GGT, and CDT, in addition to mean CDT levels for in distinct PEth intervals. Conversion factor for PEth: 1 μmol/L = 703 ng/ml.

PEth intervals (μmol/L)	No. of samples	No. of patients	Age, mean (SD), years	AST above ref. range, %	ALT above ref. range, %	GGT above ref. range, %	CDT above ref. range, %	CDT mean (SD), %-points
Total	34 483	19 958	55.3 (15.3)	10.4	11.7	14.4	9.4	1.8 (2.5)
<0.015	7837	5749	51.9 (16.6)	3.6	6.8	5.3	0.1	0.7 (.2)
0.015–0.030	1903	1739	51.9 (17.3)	4.9	7.6	5.8	0.2	0.7 (.2)
0.031–0.100	4303	3647	53.2 (16.8)	4.1	7.6	6.1	1.1	0.9 (.4)
0.101–0.200	3998	3248	55.6 (15.5)	5.6	9.3	8.5	3.5	1.0 (.6)
0.201–0.300	2998	2475	56.5 (14.8)	6.4	9.5	10.7	6.6	1.3 (.9)
0.301–0.500	3808	3000	58.1 (14.0)	8.3	11.6	13.6	11.1	1.6 (1.3)
0.501–1	4739	3490	58.2 (13.4)	12.6	14.2	21.2	18.1	2.3 (2.2)
>1	4897	3286	58.0 (11.8)	34.7	26.2	40.6	31.9	4.4 (4.3)

Table 3 presents the estimated mean levels of HDL-C from the mixed model analysis for the different PEth groups for males. Additionally, the table displays the estimated mean consumption of ethanol in each PEth group, when assuming zero consumption in the first PEth group and an expected increase in HDL-C level of .0035 mmol/L per gram of ethanol consumed per day. Samples were excluded from the calculations in the zero PEth group if they concurrently exhibited a CDT result of 1.7 percentage points or higher (N = 16), in order to ensure a more accurate classification of the zero consumption group (Årving et al. 2023).

**Table 3:** Mean HDL-C values for males (calculated estimated mean through mixed model) in distinct PEth intervals. The table also displays the corresponding estimated daily ethanol consumption in grams. This calculation was based on a .0035 mmol/L increase in HDL-C per gram of daily consumed ethanol (Rimm et al. 1999). Conversion factor for PEth: 1 μmol/L = 703 ng/ml

PEth intervals (μmol/L)	No. of samples	No. of patients	HDL-C mean (estimated mean mixed model) (mmol/L)	Estimated daily ethanol consumption (grams) Derived from HDL-C increase of .0035 mmol/L per gram of pure ethanol
<0.015	7827	5743	1.17	0
0.015–0.030	1903	1739	1.20	9
0.031–0.100	4303	3643	1.24	20
0.101–0.200	3998	3248	1.25	23
0.201–0.300	2998	2475	1.30	37
0.301–0.500	3808	3000	1.35	51
0.501–1	4739	3490	1.49	91
>1	4897	3286	1.83	189

Table 3 demonstrates that male individuals with a PEth value of zero (<.015 μmol/L, <11 ng/ml) exhibited an estimated mean HDL-C of 1.17 mmol/L, with zero ethanol consumption presumed in this group. The estimated mean HDL-C among male patients with PEth in the interval .031–0.100 μmol/L (22–70 ng/ml) was 1.24 mmol/L, which is .07 mmol/L higher than the presumed zero consumption group. This difference would correspond to a mean daily ethanol consumption of 20 grams, when .0035 mmol/L difference in HDL-C per gram ethanol is inferred. Male patients with a measured PEth in the interval .301–0.500 μmol/L (212–351 ng/ml) demonstrate an estimated mean HDL-C of 1.35 mmol/L, which would correspond to a mean daily intake of 51 grams ethanol, under the same assumptions.

**Figure 2:**
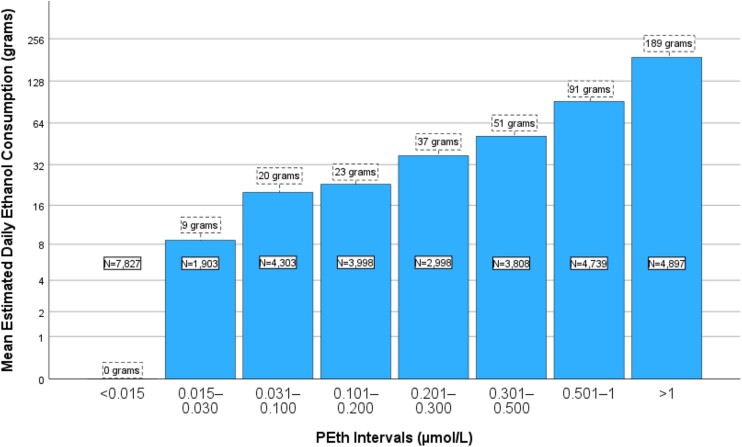
Histogram illustrating the estimated mean daily ethanol intake in grams for men across different PEth intervals. The estimated consumption values are presented in boxes with dashed lines for each interval, and the number of samples in each interval is depicted in boxes with solid lines within each column. The Y-axis is logarithmic with a base of 2.

The association between various PEth intervals and the corresponding calculated mean daily ethanol consumption is depicted in Fig. 2.

In 5.3% of the overall samples, ethanol analysis was also conducted. Excluding samples with a positive ethanol result (>.03 g/kg) (N = 266) did not yield any significant differences in the calculated mean HDL-C concentrations presented in Table 3.

## Discussion

This study investigated patients with both PEth and HDL-C measured simultaneously from one or more sampling occasions. Given the previously established correlation between increases of HDL-C levels and ethanol consumption at the population level, the mean HDL-C concentration across various PEth interval groups could provide an estimate of the corresponding mean consumption associated with different PEth concentrations. To our knowledge, this study is the first which tries to quantify the PEth–alcohol association using HDL-C as a surrogate marker for alcohol consumption, and we hope that our approach provides a novel perspective on interpreting PEth levels.

Our results suggested that male patients exhibiting PEth values in the range of .031–0.100 μmol/L (22–70 ng/ml) have an estimated mean daily ethanol consumption of 20 grams, whereas a mean daily intake of ~50 grams is estimated for those patients with PEth concentrations in the .301–0.500 μmol/L (212–351 ng/ml) interval. These two intervals represent patients with measured PEth values above the two suggested interpretation thresholds for moderate (.03 μmol/L or ~ 21 ng/ml) and heavy consumption (.3 μmol/L or ~ 211 ng/ml), respectively (Luginbühl et al. 2022).

The results of this study show a significant correlation between HDL-C and PEth values for both men and women, supporting previous research indicating a relationship between alcohol consumption and changes of HDL-C. In the review study by Rimm et al. (1999) 29 data records included male participants who increased their alcohol consumption in an experimental setting. The included studies were weighted according to number of participants, and the weighted mean increase in HDL-C was .0035 mmol/L per gram of pure ethanol increase. It is well established that HDL concentrations correlate with endogenous estrogen levels and the use of oral contraceptives, which could account for the observed higher HDL-C concentrations in samples from females (Kruszon-Moran et al. 1988).

Other studies have also explored the relationship between HDL-C and ethanol consumption. Another meta-analysis of experimental studies demonstrated a dose–response relationship, with a mean increase in HDL-C of .103 mmol/L among individuals consuming 30–60 grams of ethanol per day (Brien et al. 2011). Additionally, a recent cohort study reported a 7.1% (6.4% for men and 7.4% for women) increase in HDL-C with the consumption of one additional serving of alcohol (14 grams of ethanol) per day (Li et al. 2023). This finding exceeds our estimation, which suggests approximately a 4% increase in HDL-C for the same increase in ethanol consumption, when assuming zero consumption in the initial PEth group and an increase of .0035 mmol/L in HDL-C per gram of ethanol. It is important to note that the previous cohort study relied on self-reported alcohol intake via questionnaires, which could lead to underreporting. Moreover, the population in the cohort study from Li et al. (2023) had a higher proportion of women (N = 34 754) than men (N = 6872), differing from the experimental studies in the meta-analysis by Rimm et al. (1999), predominantly involving male participants.

Given the limitations of our study design in estimating ethanol consumption across various PEth levels, our findings should only be considered complementary to those from studies employing different methodologies. We recognize that while our approach could provide valuable insight into alcohol consumption patterns at a population level, the use as a standalone metric for individuals would be more uncertain as HDL-C is influenced by numerous factors.

Nonetheless, it is noteworthy that our results align with and bolster previous research (Piano et al. 2015; Helander et al. 2019; Jørgenrud et al. 2021; Skråstad et al. 2023; Finanger et al. 2024). Our estimation for the interval .201–0.300 μmol/L (141–211 ng/ml) in PEth was 37 grams of ethanol per day and 51 grams per day for the .301–0.500 μmol/L (212–351 ng/ml) PEth interval. A prior self-report study found a mean consumption of 33 grams of ethanol per day for binge drinkers with a mean PEth concentration of 186 ng/ml (.26 μmol/L) (Piano et al. 2015). Other studies including self-report data indicated an ethanol consumption of ~42 grams per day (Jørgenrud et al. 2021) and 35 grams per day (a median of 492 grams over the past two weeks) (Helander et al. 2019) for PEth levels to exceed .3 μmol/L (211 ng/ml). Results from a large Norwegian population study indicated a median PEth of about .3 μmol/L in blood samples from participants self-reporting a daily intake of 24–36 grams of ethanol (Skråstad et al. 2023). A recent study from the same study group, involving 62 participants, found a mean PEth concentration of .11 μmol/L for a mean daily alcohol consumption of 30 grams based on prospective self-reports, and 26 grams based on retrospective self-reports (Finanger et al. 2024). In comparison, our estimates indicate a mean daily intake of 23 grams of ethanol for individuals within the PEth interval of .101–0.200 μmol/L (71–140 ng/ml).

When considering the .03 μmol/L PEth threshold for distinguishing between low and moderate intake, previous research findings are relatively scarce, but still consistent with our results. The self-report study by Piano et al. (2015) found a mean ethanol intake of 8.3 grams per day for moderate drinkers with a mean PEth concentration of 24 ng/ml (~.03 μmol/L). Our data suggest an average consumption of ~20 grams per day among patients with PEth values falling within the .031–0.100 μmol/L (22–70 ng/ml) range. A former review study concludes with a daily consumption averaging 21–28 grams for women and ~35 grams for men to produce a PEth value exceeding 20 ng/ml (~.03 μmol/L) (Ulwelling and Smith 2018).

We employed mixed model statistics to accommodate the inclusion of multiple samples per patient. However, a limitation of these analyses is that they do not account for the sequence of multiple samples for each patient; rather, they are treated in a random order. Thus, as an alternative approach to validate our findings from these analyses, we explored the correlation between alterations in HDL-C and PEth levels across sequential samples from male patients who provided a minimum of two samples. This would elucidate the intra-individual variations in HDL in relation to PEth changes. Our selection criteria also demanded a CDT elevation of at least .2 percentage points. This stringent CDT criterion served as additional support of genuine increases in alcohol consumption between two consecutive samples (Helander et al. 2016). The ratio between the increase in HDL-C and the increase in PEth exhibited considerable variability, although the mean value provides an indication of the central tendency. Our analysis revealed a mean increase of .38 mmol/L HDL-C for every 1 μmol/L rise in PEth concentration (results not shown), which is only slightly higher than the slope for all samples observed in the linear regression equation in fig. 1.

The present study has some significant weaknesses. The primary one being that while we can estimate the central tendency of alcohol consumption in different PEth groups, we are unable to demonstrate the spread of alcohol consumption within these groups. Although we can present the spread of HDL-C concentrations, the variability in the increase of HDL-C linked to alcohol consumption cannot be quantified. The patients included in the various PEth groups represent different populations, complicating any such assessment. According to previous research, the interindividual differences in PEth levels achieved after similar alcohol consumption levels are substantial (Gnann et al. 2012). Unfortunately, this variation could not be quantified in the present study.

We also chose to base our study on the relationship between HDL-C and alcohol consumption as established in the extensive meta-analysis by Rimm et al. (1999). Given their experimental design, we deemed these studies more reliable than those relying on self-reported data (Li et al. 2023), though this assumption can be debated. We recognize that experimental studies also have limitations, particularly in generalizing findings to broader populations than the study subjects, with varied activity levels and clinical conditions which can affect the relationship between alcohol intake and HDL-C levels.

Further, we selected Rimm et al. (1999) over the more recent meta-analysis by Brien et al. (2011) because Rimm provided a specific coefficient for HDL-C response per gram of daily ethanol consumption, even though the underlying data (29 records for men) showed considerable variability. Brien’s study presented broader consumption intervals in the results, making direct estimation per gram more complex. In their discussion, however, they state an increase in HDL-C of .0032 mmol/L per gram for a 30-gram dose of ethanol. Using this alternative estimate, our data would suggest mean ethanol intake of 22 grams for the PEth range of .031–0.100 μmol/L (22–70 ng/ml) and 56 grams for the .301–0.500 μmol/L (212–351 ng/ml) range, compared to our original estimates of 20 grams and 51 grams, respectively.

Additionally, the assumption of zero consumption in patients with PEth levels below the LoQ may lack precision, even though we excluded samples with elevated CDT levels. Patients with PEth below the LoQ may still have consumed small amounts of alcohol. This assumption was made to simplify the model, as we lack a basis for determining a reference level of alcohol consumption in the first PEth group. We acknowledge that, as a result of this simplification, the estimates for the other PEth intervals at the population level may be slightly underestimated. This inaccuracy is likely to have the greatest impact on the lower PEth intervals, where the estimated consumption amounts are smaller.

Another limitation of our study is the absence of clinical information regarding the individuals from whom blood samples were obtained. We lacked data on potential medical conditions, self-reported ethanol intake and the timing of alcohol consumption in relation to blood sampling. Since PEth accumulates over time, the precise timing of drinking episodes relative to sample collection could potentially influence the observed correlations between PEth and HDL-C levels.

The strength of the current study lies in its large sample size, encompassing 50 751 samples, which hopefully helps to mitigate some of the limitations related to the lack of clinical information. Additionally, the utilization of fully validated, robust analytical methods conducted in a consistent laboratory setting for all included patients adds to its strength.

Traditional interpretations of measured PEth values typically classify a patient’s drinking behavior as e.g. either ‘social drinking’ or ‘harmful drinking.’ However, research exploring ethanol consumption and its corresponding PEth values may introduce a more precise or nuanced interpretation, providing an approximate estimate of the consumed ethanol amount in grams. The present study hopefully contributes to this.

## Conclusion

We conclude that the present study suggests that measured PEth levels above .03 μmol/L (21 ng/ml) may possibly indicate a mean ethanol consumption of ~20 grams of pure ethanol per day, while PEth levels above .3 μmol/L (211 ng/ml) may suggest a mean ethanol consumption of ~50 grams of pure ethanol per day. These estimates are derived from HDL-C measurements in male patients, and applying these findings to women requires caution due to known sex-specific differences in alcohol metabolism (Vatsalya et al. 2023) and HDL-C response (Li et al. 2023). However, the data for females indicate a similar PEth-HDL-C correlation, warranting future research into sex-specific differences in alcohol metabolism and PEth interpretation. Although the use of increase in HDL-C to estimate the amount of consumed ethanol is not an ideal design, conducting experimental studies is challenging, and the current findings could serve as a supplement to enhance the interpretation of PEth concentrations.

## Data Availability

The authors confirm that the data supporting the findings of this study can be made available from the corresponding author upon reasonable request.
